# Function and regulation of cGAS-STING signaling in infectious diseases

**DOI:** 10.3389/fimmu.2023.1130423

**Published:** 2023-02-06

**Authors:** Yang Du, Zhiqiang Hu, Yien Luo, Helen Y. Wang, Xiao Yu, Rong-Fu Wang

**Affiliations:** ^1^ Department of Medicine, and Norris Comprehensive Cancer Center, Keck School of Medicine, University of Southern California, Los Angeles, CA, United States; ^2^ Research Center of Medical Sciences, Guangdong Provincial People's Hospital (Guangdong Academy of Medical Sciences), Southern Medical University, Guangzhou, Guangdong, China; ^3^ Department of Immunology, School of Basic Medical Sciences, Southern Medical University, Guangzhou, Guangdong, China; ^4^ Department of Neurology, Guangdong Neuroscience Institute, Guangdong Provincial People’s Hospital (Guangdong Academy of Medical Sciences), Southern Medical University, Guangzhou, Guangdong, China; ^5^ Department of Pediatrics, Children’s Hospital, Keck School of Medicine, University of Southern California, Los Angeles, CA, United States; ^6^ Guangdong Provincial Key Lab of Single Cell Technology and Application, Southern Medical University, Guangzhou, Guangdong, China

**Keywords:** cGAS, STING, infectious diseases, immune regulation, innate immunity

## Abstract

The efficacious detection of pathogens and prompt induction of innate immune signaling serve as a crucial component of immune defense against infectious pathogens. Over the past decade, DNA-sensing receptor cyclic GMP-AMP synthase (cGAS) and its downstream signaling adaptor stimulator of interferon genes (STING) have emerged as key mediators of type I interferon (IFN) and nuclear factor-κB (NF-κB) responses in health and infection diseases. Moreover, both cGAS-STING pathway and pathogens have developed delicate strategies to resist each other for their survival. The mechanistic and functional comprehension of the interplay between cGAS-STING pathway and pathogens is opening the way for the development and application of pharmacological agonists and antagonists in the treatment of infectious diseases. Here, we briefly review the current knowledge of DNA sensing through the cGAS-STING pathway, and emphatically highlight the potent undertaking of cGAS-STING signaling pathway in the host against infectious pathogenic organisms.

## 1 Introduction

The innate immune system serves as the first line of host defense against infectious diseases by detecting the pathogen-associated molecular patterns (PAMPs) of the pathogens. This task relies on the germline-encoded pattern recognition receptors (PRRs), including Toll-like receptors (TLRs), RIG-I-like receptors, Nod-like receptors (NLRs), C-type lectin receptors and intracellular DNA and RNA sensors (1–3). Upon detection of PAMPs from pathogens, signaling cascades through these receptors lead to the production of inflammatory cytokines and chemokines, as well as the initiation of cell death to eliminate infected cells (3–5).

Sensing the microbial DNA, best known as the blueprint of life, acts as a key element in triggering host defense, and spans across a wide variety of species. In mammalian cells, three major innate immune receptors have been identified as responsible for DNA sensing, including TLR9, absent in melanoma 2 (AIM2), and cyclic GMP–AMP synthase (cGAS). TLR9 localizes on the endosomal membrane and recognizes single-stranded DNA (ssDNA) specifically containing unmethylated cytidine-phosphate-guanosine (CpG) motifs from bacteria or viruses (6, 7). AIM2 contributes to the recognition of double-stranded DNA (dsDNA) in the cytosolic compartment through the formation of inflammasomes, inducing the activation of cysteine-aspartic proteases 1 (Caspase 1), resulting in the proteolytic maturation of the pro-inflammatory cytokines interleukin-1β (IL-1β) and IL-18, as well as cell pyroptosis (8–13). Recently, dsDNA sensing, which is mediated by cyclic-GMP-AMP (cGAMP) synthase (cGAS)-stimulator of interferon genes (STING) pathway, has been considered as a crucial element for linking the recognition of pathogens DNA to the establishment of host innate immune defense state (1, 14, 15). Upon binding to dsDNA, the catalytic activity of cGAS is triggered, leading to the production of 2’3’-cGAMP, which acts as the secondary messenger that subsequently binds and activates the adaptor protein STING on the endoplasmic reticulum (ER) membrane, and mediates downstream pathway to produce an array of type I and type III interferons (IFNs) (16, 17). The existence of homologs of cGAS and/or STING in various organisms and the sequence-independent manner of sensing dsDNA from pathogens confer cGAS-STING signaling pathway a powerful role in the innate immune system (18). In this review, we briefly summarize the current understanding of DNA sensing through the cGAS-STING pathway and mainly focus on the potent capacity of cGAS-STING signaling pathway in the host immunity against infectious diseases.

## 2 cGAS-STING pathway

DNA is mostly squeezed in the nucleus and mitochondria and expeditiously degraded by cytosolic and lysosomal DNA-degrading nucleases in normal circumstances (19). Upon infections, increased amounts of foreign intracellular DNA are sensed by cGAS (20, 21), which belongs to the nucleotidyltransferase (NTase) enzyme family (22). cGAS is composed of a C-terminal NTase domain and an N-terminal highly positively charged domain (21, 23–25). cGAS binds to dsDNA in a sequence-independent manner. Upon interacting with DNA, the catalytic domain of cGAS undergoes a structure conformational change, which induces the synthesis of 2’3’-cGAMP from adenosine triphosphate (ATP) and guanosine triphosphate (GTP) (24, 26, 27). Although cGAS could bind with short dsDNA (~20 bp), it prefers longer length of DNA (>45 bp), which could facilitate cGAS to form more stable dimers and trigger a stronger enzymatic activity (28, 29). Notably, ssDNA could also interact with cGAS (30–32). However, such binding cannot lead to the activation of cGAS, suggesting precise enzymatic activation of cGAS by dsDNA. Besides the C-terminal NTase domain, the N-terminal domain is reported to facilitate the synthesis of 2’3’-cGAMP by increasing the enzymic activation of cGAS *via* induction of cGAS-DNA liquid–liquid phase separation (29). The occurrence of such phase separation is dependent on a high concentration of cGAS and DNA (each exceeded 30 nM) (29), illustrating that only when the amount of cytosolic DNA reaches a sufficient level, cGAS is activated. This critical threshold of activation of cytosolic cGAS by foreign intracellular DNA renders a proper safeguard mechanism for the host. However, in the case of exposure to self-DNA, cGAS can cause auto-inflammation and pathology (31, 33–36). Besides cytoplasm, cGAS is also present in the nucleus which is rich in genomic DNA (37, 38). The nuclear localization of cGAS relies on a structurally more accessible cGAS catalytic domain which is also necessary for cGAS tethering to chromatin (38). Based on this, emerging studies have focused on why nuclear cGAS is unable to be activated by self-genomic DNA. In 2020, several groups employed Cryo-EM and revealed 11 structures of cGAS bound to the nucleosome in total, and these interactions are mainly dependent on histones H2A/H2B, which suggested how cGAS is tethered to and suppressed by chromatin inside nuclear and thus prevent autoreactivity (39–44). Consistently, Li et al. reported that cGAS activity could be suppressed *via* hyperphosphorylation at the N terminus and inhibition of oligomerization due to chromatin tethering during mitosis, which could prevent cGAS phase separation into liquid droplets to synthesize cGAMP (45). Generally, these findings identify cGAS as a vital guard in maintaining cell homeostasis and defending against danger from outside.

2’3’-cGAMP, then binds to STING, on the ER membrane (16, 26, 46). STING contains an N-terminal transmembrane segment, and a cytoplasmic ligand-binding domain (LBD), followed by a C-terminal tail (CTT) (47–51). In the absence of a ligand, STING forms a homo-dimer through two CTTs, exhibiting as a V-shaped binding pocket facing the cytosol. In the presence of cGAMP, cGAMP binding leads to the closure of the ligand-binding pocket and subsequently triggers a 180° rotation of LBD relative to the transmembrane domain. This rotation is accompanied by a conformational rearrangement on the side of the LBD dimer, inducing the formation of the STING tetramer and higher-order oligomers (51). This ligand-induced oligomerization then triggers the translocation of STING from ER to ER-Golgi intermediate compartment (ERGIC) and the Golgi (46, 52). The ER-to-Golgi traffic is dependent on the GTPase SAR1A, coat protein complex-II (COP-II), and ADP-​ribosylation factor (ARF) GTPases (53, 54). So far, what signal within STING is sensed to regulate this trafficking process remains poorly understood. It is reported that STING is retained on the ER membrane by Ca^2+^ sensor stromal interaction molecule 1 (STIM1), restricting the activation of STING *via* its translocation from the ER to the ER-Golgi intermediate compartment (55). On the contrary, STEEP facilitates STING ER exit through elevating phosphatidylinositol-3-phosphate (PtdIns(3)P) production and ER membrane curvature formation (56). Recently, abnormal Golgi-to-ER STING retrieval is reported in COPA syndrome, which is characterized by chronic up-regulation of type I IFN signaling. Further mechanism study shows that mutations in *COPA* result in the accumulation of ER-resident STING at the Golgi, leading to enhanced type I IFN signaling (57).

**Figure 1:**
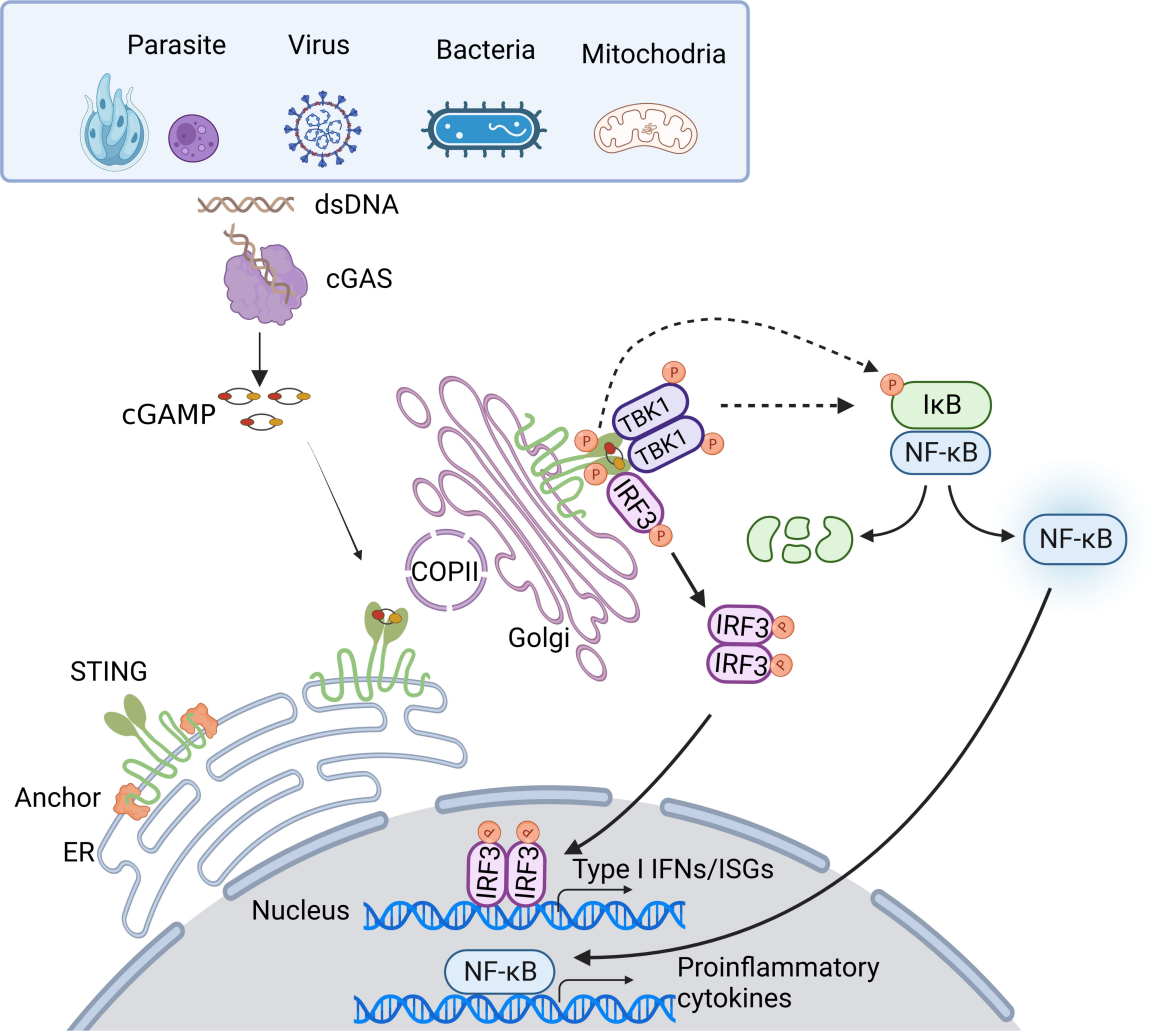
Overview of the cGAS-STING DNA-sensing pathway. Cytosolic DNA sensing by cGAS-STING pathway is implicated in pathogen infections. DNA from pathogens or infections-induced mitochondria release can enter the cytosol and initiate the cGAS-STING pathway. Upon binding dsDNA, cGAS is activated and produces 2’3’-cGAMP. cGAMP, the secondary messenger, subsequently binds STING, leading to conformational rearrangement, which induces the formation of the STING tetramer and higher-order oligomers release from anchor proteins, and translocation from the ER to Golgi *via* COPII. At Golgi, STING oligomers recruit TBK1, triggering TBK1 autophosphorylation. In turn, STING is phosphorylated by TBK1. Phosphorylated STING then recruits IRF3, facilitating the phosphorylation of IRF3 by TBK1. Activated IRF3 dimers then translocate to the nucleus to activate the transcription of type I IFNs, which in turn leads to the production of diverse IFN-stimulated genes (ISGs). Besides, STING induces NF-κB activity *via* TBK1-dependent and independent manners, resulting in the IκBα phosphorylation and subsequently translocation of NF-κB to the nucleus, leading to the production of inflammatory cytokines.

Once trafficking to the Golgi compartments, STING recruits TANK-binding kinase 1 (TBK1) to initiate downstream signaling (16). STING is known to undergo diverse post-translational modifications, including phosphorylation, palmitoylation, ubiquitination, SUMOylation, nitro-alkylation, glycosylation, carbonylation, reversible oxidation, and et al. (58–60). Palmitoylation of STING at the trans-Golgi network (TGN) is essential for the activation of STING. Mutation at cysteine residues 88/91 (Cys88/91) attenuates the palmitoylation and abolishes the induction of type I IFN response (61). In addition, covalent small-molecule inhibitors that target the Cys91 block the activation-induced palmitoylation of STING, which is essential for the clustering of STING at the Golgi apparatus and, in turn, for the recruitment of TBK1 (62). Following STING trafficking to the Golgi compartments, STING oligomers recruit TBK1 through the conserved PLPLRT/SD amino acid binding motif within CTT, which triggers the activation of TBK1 *via* dimerization-mediated TBK1 autophosphorylation at Ser172, a residue that is important for TBK1 activation (63–65). In turn, STING is phosphorylated by TBK1 at Ser366 within CTT, which is part of pLxIS (p, hydrophilic residue; x, any residue; S, phosphorylation site). This phosphorylated STING then recruits IRF3, facilitating the phosphorylation of IRF3 by TBK1 (66). Notably, either S366A STING or L374A STING abrogates the interaction between STING and IRF3, and subsequent phosphorylation of IRF3 by TBK1 (65). Activated IRF3 dimers then translocate to the nucleus to activate the transcription of type I IFNs, which in turn leads to the production of diverse IFN-stimulated genes (ISGs), establishing a robust anti-infection state (67). Besides type I IFN signaling cascades, STING also activates nuclear factor-κB (NF-κB) through a poorly discovered mechanism. Emerging evidence suggests STING induces NF-κB activity *via* a TBK1-independent mechanism, which is distinct from IRF3 activation. NF-κB activation through the miniCTT (subdomain in the C-terminal domain), distinct from the TBK1 binding domain, suggests the activation is not fully dependent on the CTT, which rules out a role for TBK1 (68). Blocking K224 and K288 ubiquitination of STING strongly impairs IRF3 but not NF-κB activation. Mechanism studies show that K224 and K288 on STING are critical for trafficking from ER to Golgi, illustrating that STING may activate the NF-κB pathway prior to its trafficking to the Golgi compartment for TBK1 interaction (69, 70). Besides, TBK1 is dispensable for NF-κB activation downstream of STING in myeloid cells. TBK1 acts redundantly with IκB kinase ε (IKKε, also known as IKKi) to induce NF-κB upon STING activation. Notably, TAK1 and IKKβ are essential for STING-induced-NF-κB signaling. A study in zebrafish shows that the conserved PxExxD motif at the CTT of zebrafish STING could directly associate with tumor necrosis factor receptor associated factor 6 (TRAF6) to trigger NF-κB signaling (71). In addition, in an etoposide-induced DNA damage context, STING-mediated NF-κB activation relies on TRAF6-dependent K63-linked ubiquitylation of STING, in which process involves interferon-γ-inducible factor 16 (IFI16) and tumor suppressor p53, and is independent on TBK1 (72), suggesting in certain contexts, STING may dictate cytokine production through alternative routes. Taken together, the cGAS-STING pathway utilizes diverse downstream factors to eliminate intracellular pathogens (**Figure 1**).

## 3 cGAS-STING in parasitic infectious diseases

**Figure 2:**
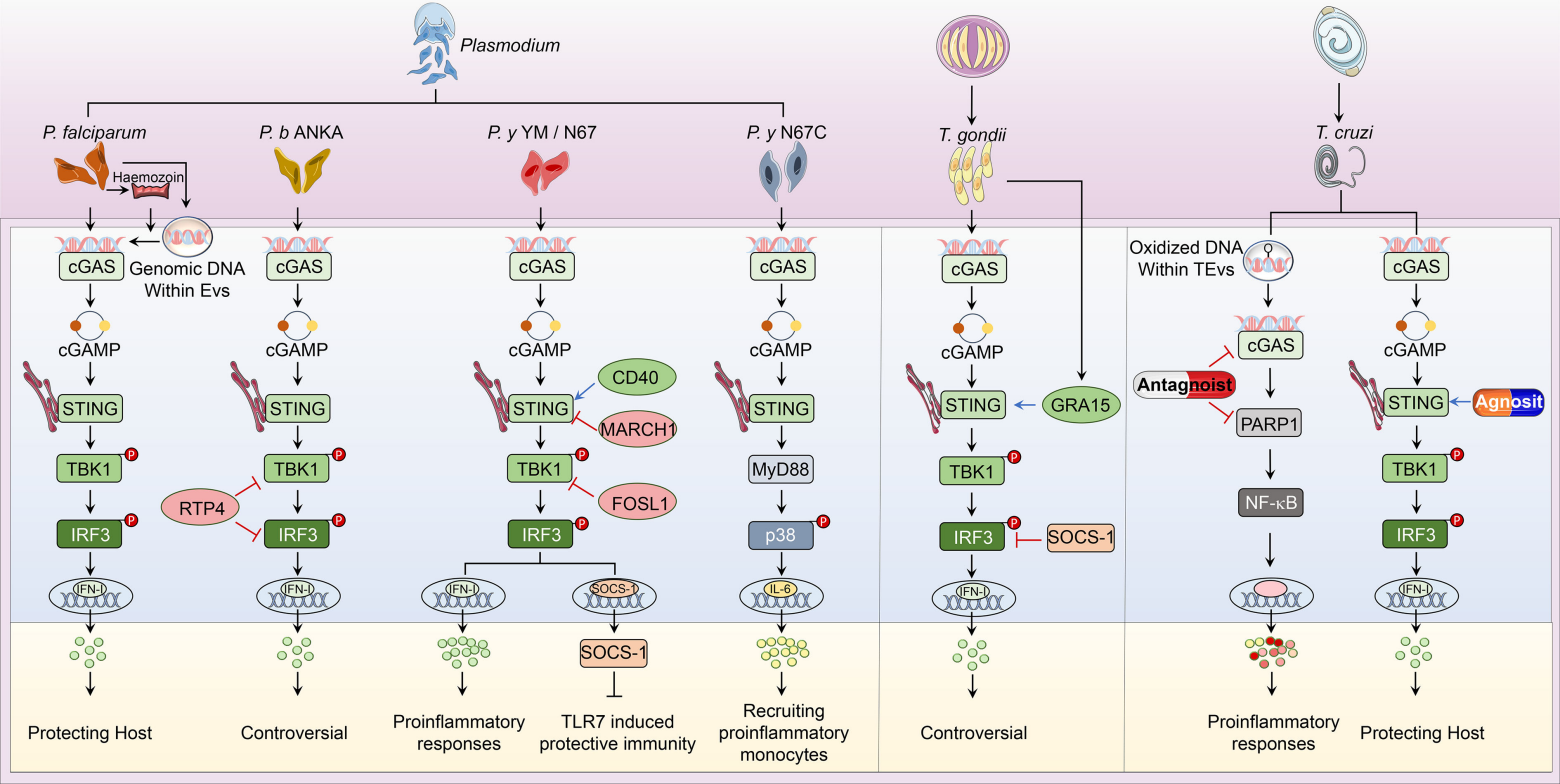
Overview of cGAS-STING pathway in host defense against parasitic infection. *i. The classical cGAS-STING-type I IFN pathway.* cGAS is an innate immune sensor that recognizes a diverse array of parasitic genomic DNA of *Plasmodium*, *T. gondii*, and *T. cruzi*, as well as EVs containing *P. falciparum*. Upon binding parasitic gDNA, cGAS oligomerizes with these gDNAs and performs its catalytic role to synthesize 2’3’ cGAMP. cGAMP then binds to STING at the ER membrane and stimulates the oligomerization of STING. Following, STING translocates from the ER to Golgi and recruits TBK1, which induces TBK1 autophosphorylation. Activated TBK1 phosphorylates STING, which in turn recruits IRF3 for TBK1-mediated phosphorylation. Phosphorylated IRF3 dimers translocate to the nucleus and trigger type I IFN production, which determines the established or disputed disease outcomes. *ii. The nonclassical cGAS-STING pathway.* During *P. y* YM infection, cGAS-STING signaling induces a negative regulator SOCS1 expression to inhibit TLR7-MyD88-mediated type I IFN responses in pDCs. For *P. y* N67C infection, cGAS-STING-MyD88-p38 signaling specifically induces late IL-6 production, which expands CD11b^+^Ly6C^hi^ proinflammatory monocytes to inhibit immunity. Upon *T. cruzi* infection, EVs containing *T. cruzi* gDNA activate cGAS and trigger the PARP1-dependent NF-κB signaling, which induces proinflammatory responses. *iii. Regulators of cGAS-STING pathway.* RTP4, acting as a negative regulator, targets both TBK1 and IRF3 during *P. b* ANKA infection. Upon *P. y* YM/N67 infection, MARCH1 and FOSL1 negatively adjust STING and TBK1 respectively. Besides, CD40 is a positive regulator of STING-promoting type I IFN responses. For *T. gondii* infection, SOCS1 interacts with IRF3 to suppress cGAS-STING-type I IFN signaling, but GRA15 secreted from this parasite enhances type I IFN responses by activating STING. At the phase of *T. cruzi* infection, cGAS or PARP1-specific antagonists have been used to suppress cGAS-PARP1-NF-κB signaling, which limits harmful proinflammatory responses. Besides, vaccines containing STING agonists could also enhance anti-*T. cruzi* immunity by promoting type I IFN signaling.

Parasitic diseases, caused by worms and protozoa, are one of the common infectious zoonoses that severely threaten host health. Anti-parasite innate immunity is the key weapon for the host defense against parasitic invasion, which also decides the end of parasitosis’s clinical outcome. In the process of parasite invasion, DNA released from pathogens is sensed by multiple nucleic acid sensors. Among them, cGAS-STING signaling is the most widely studied immune pathway participating in innate immunity against *Plasmodium*, *Toxoplasma gondii* (*T. gondii*), *Trypanosoma cruzi (T. cruzi*), and *Schistosoma mansoni* (*S. mansoni*) infection (**Figure 2**).

### 3.1 Malaria

Malaria caused by *Plasmodium* infection is a deadly infectious disease that affects 247 million people worldwide; it resulted in about 619,000 global deaths in 2021 [WHO, 2022]. During malaria infection, multiple *Plasmodium* biological products, such as GPI-anchors (73–77), heamozoin (78–82), genomic DNA (gDNA) (78, 83–88) and RNA (87, 89, 90), are acting as PAMPs recognized by host PRRs existing in innate immune cells including monocytes, macrophages, conventional dendritic cells (cDCs) and plasmacytoid dendritic cells (pDCs) (84, 91–96). Among these PAMPs-PPRs interactions, cGAS is a non-negligible sensor sensing *Plasmodium* gDNA in addition to TLR9. Early studies revealed *Plasmodium* gDNA, which is rich in AT-motif (97), could induce type I IFN responses *via* an uncharted DNA receptor but not these well-known sensors like TLR9, DAI, RNA polymerase-III or IFI16/p204 (86). To explore this hidden character recognizing AT-rich DNA, we built a *Plasmodium yoelii* (*P. y*) YM infection mouse model using *Mb21d1^–/–^* (coding for cGAS), and *Tmem173^gt^* mice. *Tmem173^gt^* is an I199N missense mutant allele of the STING gene (formerly, *Tmem173*). *Tmem173^gt^* mice cannot produce IFN-β in response to cyclic dinucleotides (98). In line with our *in vitro* study, we found that cGAS functions as a DNA sensor in pDCs for detecting YM gDNA at the early infection stage and primarily induces STING-mediated type I IFN signaling (99). Consistently, by using human monocytes, another research group also identified cGAS as the cytosolic sensor of *P. falciparum* (*P. f.*) gDNA, and access of parasitic gDNA to the cytosolic compartment was mediated by *Plasmodium* hemozoin (100). In addition, extracellular vesicles (EVs) secreted by *P. f.* infected RBCs are confirmed as a vehicle transferring parasitic genomic DNA to immune cells, which then stimulate STING-TBK1-IRF3-dependent gene induction and type I IFN production (101). Overall, these researches indicate that cGAS is essential for *Plasmodium* gDNA detection and STING-triggered type I IFN responses during malaria.

Although cGAS-STING mediated activation of type I IFN signaling is solid, the effects of cGAS-STING signaling on the pathological outcome of *Plasmodium*-infected hosts are still controversial (102). With regards to the role of type I IFN in *P. falciparum* infection, several clinical studies have indicated that children with mild malaria show a higher IFN-α level than those with severe malaria (103). Lower circulating IFN-α is also observed in children from Kenya with severe malaria anemia (SMA). Two polymorphisms [IFN-α2 (A173T) and IFN-α8 (T884A)] in IFN-α promoter regions, which reduce IFN-α generation, is related to increased susceptibility to SMA (104). As for the mouse malaria model, we firstly reported activation of cGAS-STING, and MDA5-MAVS by *P. y* YM infection, triggered IRF3-mediated limited production of IFN-α/β, which then induced negative regulator SOCS1 inhibit RNA-TLR7-MyD88-dependent more puissant type I IFN responses (99). Furthermore, we found SOCS1 expression is markedly reduced in *Ifih1* ^-/-^, *Mavs* ^-/-^, and *Tmem173* ^gt^ pDCs, allowing the activation of robust MyD88-dependent type I IFN production, which reveals a detrimental role of cGAS-STING signaling during YM infection (99). Consistently, Spaulding et al. also reported robust type I IFN production during YM infection requires priming of pDCs by activated CD169^+^ macrophages upon STING-mediated sensing of parasites in the bone marrow, and this type I IFN response causes severe disease outcomes (105). Although both groups show that cGAS-STING mediated signaling at the early YM infection stage is harmful to the host, the roles of STING in triggering type I IFN expression are quite different, which need to be further investigated. Recently, by using a lethal *P. y* N67C model, we further demonstrated the detrimental role of cGAS-STING signaling. The cGAS/STING activation recruits myeloid differentiation factor 88 (MyD88) and specifically activates the p38-dependent signaling pathway to produce late IL-6, which expands CD11b^+^Ly6C^hi^ proinflammatory monocytes to inhibit anti-malaria immunity (88). However, when it comes to *P. b* ANKA infection, STING plays a unique disputable role due to the controversial function of type I IFN. Sharma et al. reported that STING is harmful to the host as it induced TBK1-IRF-type I IFN signaling, causing the short survival of the host during *P. b* ANKA infection (86), but we recently found rescuing the STING-induced type I IFN could lower parasitemia and alleviate neurologic symptoms (106). Generally, these findings establish that cGAS-STING-induced type I IFN accelerates inflammation pathology and promotes host mortality.

However, in some cases, cGAS-STING signaling enhances immunity in some non-lethal malaria models. Hahn et al. reported *P. y* 17XNL infection leads to a protective type I IFN response in WT control, while *Mb21d1^–/–^* and *Tmem173^gt^* mice hold higher parasitemia and worsened outcomes (107). They further compared the survival of *Mb21d1^–/–^* mice between lethal and nonlethal *P. y* infections and reached a coincident conclusion that cGAS-STING is harmful to YM-infected mice (107). Generally, cGAS-STING is a double edge sword for the host to fight against malaria, which strongly relies on parasite strains and host models.

Given the importance of cGAS-STING and downstream signaling in anti-malaria immunity, emerging studies have focused on the regulation of this pathway (108). We have indicated that upon *P. y* N67 or *P. y* YM infection, mice deficient in MARCH1 had significantly better survival rates than WT mice, which indicated the negative function of MARCH1 in generating protective immunity (87). We further showed that MARCH1 could interact with STING and lower its protein level (109). In addition to MARCH1, CD40 is another vital positive regulator we reported helping to defend against malaria as the increase of CD40 expression caused by *P. y* N67 infection could enhance the protein level of STING, which in turn enhances the type I IFN production at the early stage of infection and prolongs host survival (110). Mechanistically, CD40 induced by iRBCs, parasite DNA/RNA, and various TLR ligands could compete with STING to bind TRAF2/3 and/or TRAF6 to weaken the ubiquitination of STING, which promotes the stability of STING (110). Displaying as a key downstream molecule of cGAS-STING signaling, TBK1 is the central kinase to activate type I IFN (111, 112). Activated TBK1, in turn, phosphorylates STING, which allows the STING-TBK1 complex to recruit and phosphorylate IRF3, thus inducing type I IFN and ISGs expression (5, 66). We recently suggested that the type I IFN could promote RTP4 expression post *P. b* ANKA infection (106). Moreover, RTP4 specially binds to TBK1 and inhibits type I IFN response by inhibiting TBK1 and IRF3 expression and activation thereby weakening type I IFN production (106). Inhibition of RTP4 expression may help lower parasitemia and be beneficial to alleviate symptoms of cerebral malaria (CM) and other neuropathology (106). Besides RTP4, we also discovered FOSL1 as another suppressive regulator function on TBK1 through resisting the formation of TBK1/TRAF3/TRIF (TRIF, Toll/4ll-1 Receptor-domain-containing adapter-inducing interferon-beta) complexes by limiting K63 ubiquitination of TRAF3 and TRIF and finally leads to suppression of type I IFN production at early infection and liver stages, which eventually results in *P. y* N67 parasite fast growth and host death (113). Obviously, the function of these regulators targeting STING depends on the particular role of STING in different malaria models. Although many efforts have been paid for studies about the regulation of cGAS-STING, there are still lots of research gaps to be explored for the potential value of this signaling pathway in the prophylaxis and treatment of malaria.

### 3.2 Toxoplasmosis

*Toxoplasma gondii* (*T. gondii*) is an obligate intracellular eukaryotic parasite from the phylum Apicomplexa that infects up to one-third of the global population (114). Although it normally only causes mild illness in healthy hosts, toxoplasmosis is a common opportunistic infectious disease with high mortality in individuals who are immunocompromised like AIDS patients (115). It has been reported that specific *Toxoplasma* strain–host combinations may lead to detrimental outcomes during pregnancy (116).

Innate immunity acts as the first line of host defense and observes pathogen infection, which is essential for resisting *T. gondii* infection. Particularly, cGAS-STING is the considerable immune signaling devoted to anti-*T. gondii* innate immunity. Wang et al. initially employed mice deficient in cGAS or STING to explore their role in a mouse toxoplasmosis model and found that cGAS is necessary for the activation of anti-*T. gondii* immune signaling. Consistently, STING knockout mice are much more susceptible to *T. gondii* infection than WT mice due to the impaired protective type I IFN production (117). Interestingly, they also found that mice deficient in STING exhibited more severe toxoplasmosis symptoms than cGAS-deficient mice, which suggests that there might be some other sensors getting involved in the activation of STING (117). Besides, STING could protect the host from toxoplasmosis in another IDO1-dependent way. During toxoplasmosis, AKT phosphorylated STING forms a heterodimer with TICAM2 to promote IRF3-dependent transcription of indoleamine-pyrrole-2,3-dioxygenase-1 (IDO1), which is a critical kinase limiting parasite replication (118). However, some studies exhibited an opposing role of IRF3 that promotes the replication of *T. gondii* by using *Irf3* ^-/-^ mice and IRF3 knockout cells. They found that ISGs rather than type I IFN induced by parasite-activated IRF3 were indeed essential. Moreover, they defined parasite-IRF3 signaling activation (PISA) as a novel pro-parasitic signaling pathway, which is dependent on the cGAS-STING-TBK1 signaling and requires the adaptor TRIF (119, 120). Recently, we found that type I IFN induced during toxoplasmosis was harmful to the host by promoting PD-1 expression in T cells and destroying its function on secreting IFN-γ, but the underlying mechanism of how type I IFN generated needs to be further investigated (121).

The regulation of cGAS-STING during *T. gondii* infection can be normally divided into two types: the first regulation is induced by parasitic effectors, while another one is mediated by intrinsic regulators. Wang et al. found the *T. gondii* dense granule protein GRA15 enhanced STING polyubiquitination at Lys337 and promoted STING oligomerization in a TRAF-dependent manner, and they also verified GRA15^-/-^ *T. gondii* was more virulent resulting in higher mortality of WT mice (117). A recent report expounded that *T. gondii* virulence factor TgROP18I can not only interact with IRF3 and inhibit IFN-β production, but also suppress the recruitment of ubiquitin, p62, and LC3 to the parasitophorous vacuole membrane (PVM) thus deactivating the autophagic inhibition of *T. gondii* proliferation (122). Furthermore, some intrinsic regulators can be induced during toxoplasmosis and act on cGAS-STING signaling. Gao et al. found that FAF1 expression level was downregulated by *T. gondii* infection *via* a PI3K/AKT dependent manner, which is correlated with enhanced IRF3 transcription activity. They further found that inhibiting PI3K/AKT pathway was essential for IRF3 nuclear translocation to activate the transcription of ISGs, thereby facilitating *T. gondii* proliferation (123). Caspase-1-induced IL-1β is beneficial to protecting the host against *T. gondii* infection, we recently found that IL-1/IL-1R signaling could improve the expression of SOCS1 which could interact with IRF3 to restrain type I IFN production and maintain T cell function (121). Hence, any effort to control and eradicate toxoplasmosis needs a better understanding of the regulation of innate immune responses to *T. gondii* infection, which is also required for the development of new effective vaccines and drugs.

### 3.3 Chagas disease

*Trypanosoma cruzi* (*T. cruzi*) is another zoonotic protozoan parasite, which is the etiological agent of American trypanosomiasis, or Chagas disease, and is transmitted when the infected feces of the triatomine vector are inoculated through a bite site or an intact mucous membrane of the mammalian host (124, 125). *T. cruzi* infection is lifelong in the absence of effective treatment. The most important consequence of *T. cruzi* infection is cardiomyopathy, which occurs in 20% to 30% of infected persons (126). Similar to other protozoons, the surface of *T. cruzi* is decorated with a variety of PAMPs, which are mainly composed of glycosylated molecules that are attached to the cell membrane *via* a glycosylphosphatidylinositol (GPI) anchor, acting as potent inducers of immune response (127). Except for the GPI-induced innate immunity, Choudhuri et al. recently revealed oxidized DNA encapsulated by *T. cruzi*-induced extracellular vesicles (TEvs) is an important PAMP sensed by cGAS rather than TLR9, which was necessary for PARP1-dependent NF-κB activation and proinflammatory macrophages response leading to severe chronic inflammatory pathology in Chagas disease (128). Furthermore, these researches showed short-term treatment with cGAS antagonists or PARP1 inhibitors was sufficient to potently suppress TEv-induced cytokines’ expression in macrophages, which offered a potential therapy for controlling Chagas disease (128). However, a more recent study identified STING signaling as a pivotal role in splenic IFN-β and IL-6 expression at early infection (129). STING deficiency led to a weakened production of splenic parasite-specific IFN-γ as well as a reduction of IFN-γ/perforin-producing CD8^+^ T cells (129). Though this protective role of STING signaling was recently proposed, studies concentrating on STING agonists applied for the treatment of Chagas disease were first reported by Malchiodi’s group in 2017. Initially, this group found STING agonist c-di-AMP is an efficient adjuvant, which couples with Tc52, the parasitic antigen, could induce stronger Th17 plus Th1 specific cellular and humoral immune responses against *T. cruzi* infection (130). Subsequently, they employed c-di-AMP combined with another engineered chimeric parasitic antigen Traspain as a novel vaccine, which showed a strong protection during the whole course of the infection (131). In a recent study, they demonstrated that CpG/c-di-AMP as adjuvants could contribute to T cell priming and polyfunctional CD4^+^ and CD8^+^ T cell-mediated anti-*T. cruzi* immunity (132). Overall, these studied profoundly demonstrate that cGAS-STING acts as a protagonist of innate immunity against *T. cruzi* infection, and the vaccines formulated based on this signaling may be effective in the Chagas disease treatment.

### 3.4 Strategies for parasitic pathogens counteract cGAS-STING signaling

Although some efforts have been made to explore the vital role of cGAS-STING signaling and the related regulation, strategies for parasitic pathogens counteracting cGAS-STING signaling are still less known. Regulators like host MARCH1, RTP4, SOCS1, and FOSL1, as well as pathogen TgROP18I that we discussed above are capable of suppressing cGAS-STING signaling during parasitic infections (99, 106, 109, 113, 121, 122). However, whether there are some other host or parasitic proteins responsible for pathogens to control cGAS-STING signaling in the manner of camouflaging cytosolic foreign DNA ligands, post-translational modification of cGAS-STING signaling molecules, degradation of the 2’3’-cGAMP, or some other means still needs further studied.

## 4 cGAS-STING in viral infectious diseases

**Figure 3:**
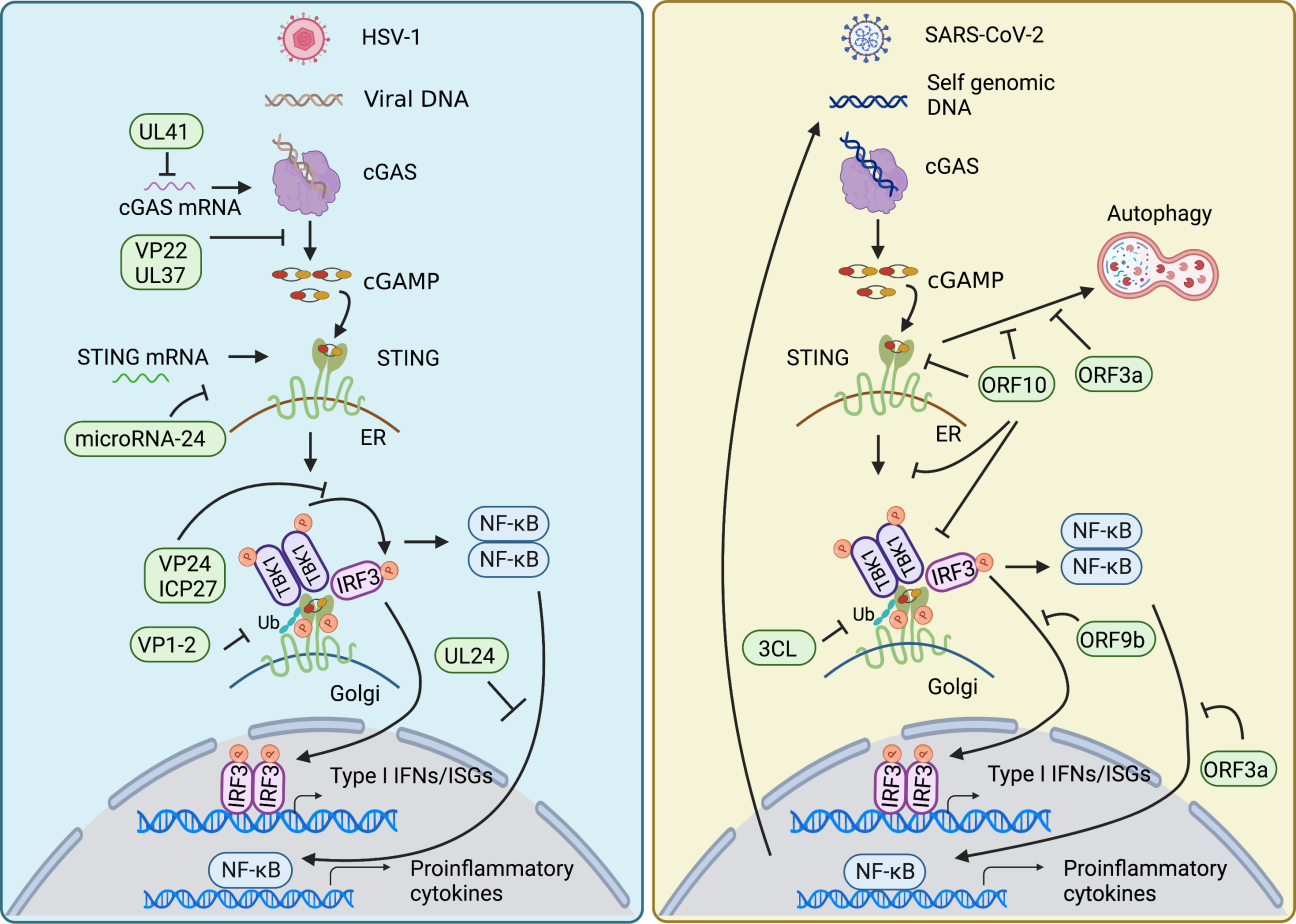
Viral evasion strategies in cGAS-STING pathway. HSV-1 and SARS-CoV-2 have developed diverse strategies to counteract cGAS-STING axis for their survival. Both mainly target signal transduction processes of the cGAS-STING pathway, such as decreasing the mRNA level of cGAS, damping the enzymatic activity of cGAS, impairing the translation of STING, restricting the translocation of STING from ER to Golgi, affecting the modification of STING, inhibiting the assembly of TBK1-activated STING signalosome, as well as nuclear translocation of NF-κB factor p65 and IRF3. In addition, SARS-CoV-2 also employs its encoding protein to intervene in STING-mediated autophagy to facilitate its replication.

Innate immunity is essential to control viral infection at the early stage of host antiviral immunity. Viral infection triggers cGAS-STING signaling pathway that induces activation of type I IFN and NF-κB signaling to restrict viral infection and to sustain homeostasis. The feature that cGAS senses DNA in a sequence-independent manner enables cGAS to detect a broad range of intracellular viral nucleic acids, thus rendering a powerful role of cGAS in the anti-viral immune response. On the other hand, viruses have developed diverse strategies to counteract cGAS-STING axis for their survival (**Figure 3**).

### 4.1 Herpes simplex

Herpes simplex virus 1 (HSV-1), a member of the alphaherpesvirus family, is an enveloped virus with a linear dsDNA genome encoding at least 84 proteins. HSV-1 infection causes a variety of pathologies, ranging from benign cold sores to fatal encephalitis (133). Several reports demonstrated that ablation of cGAS or STING rendered mice sensitive to HSV-1 infection due to the deficiency of type I IFN production (46, 134, 135), suggesting the pivotal role of cGAS-STING-IFN axis during HSV-1 infection. Furthermore, autophagy induction *via* cGAMP-mediated STING’s trafficking from ER to Golgi is shown to be crucial for clearing HSV-1 in the cytosol of infected cells *in vitro* (54), implying that STING may contribute to antiviral responses in a type I IFN-independent manner. STING-S365A mice are shown to be resistant to HSV-1 infection (136–138). Since phosphorylation of serine 365 (S365) at CTT of STING by TBK1 is required for the recruitment of IRF3, which is essential for the phosphorylation of IRF3 and induction of type I IFNs (63, 65), the antiviral response in STING-S365A mice suggests the existence of type I IFN-independent mechanism. Among these findings, one study showed that the type I IFN-independent antiviral response is contributed by STING-induced, TBK1-dependent autophagy. CTT deficiency renders the mice incapable of autophagy induction, and susceptible to HSV-1 infection (137). Whereas another group observed that L373A STING mice, which lacked TBK1/IKKε activation but preserved autophagy induction were still susceptible to HSV-1 infection, implying that autophagy alone is not enough to power the antiviral response by STING. Moreover, they suggested that the elevated transcription of NF-κB-driven genes (CXCL1 [C-X-C Motif Chemokine Ligand 1], CXCL2, and 4-1BBL [4-1BB ligand]) in the cells from STING-S365A mice may contribute to viral resistance, indicating that NF-κB is a strong candidate for the IFN-independent STING-induced antiviral responses (138).

Although the host has evolved the delicate antiviral responses, such as inductions of type I IFN, NF-κB, and autophagy, it still could lead to a lifelong latent infection in the trigeminal ganglia, due to its ability to counteract the host’s innate antiviral response (139–141). HSV-1 tegument protein UL41, an endoribonuclease with the activity of mRNA-specific RNase, has been shown to reduce the mRNA level of cGAS, thus lowing the protein level of cGAS, and subsequently decreasing cGAS/STING-mediated IFN-β production (142). Besides the mRNA level, the enzymatic activity of cGAS is inhibited by tegument protein VP22 (143). Meanwhile, HSV-1 tegument protein UL37 deamidates human and mouse cGAS, restricting the ability of cGAS to catalyze cGAMP synthesis, which is important for downstream immune response. Notably, the deamidation site is not conserved in non-human primates, implying that HSV-1 utilizes species sequence variation to counteract host defenses (144). To antagonize the STING-mediated immune response, HSV-1 VP1-2 directly deubiquitinates STING and impairs its downstream signaling (145). In addition, HSV-1 VP24 and ICP27 intervene in the association between TBK1 and IRF3 and the TBK1-activated STING signalosome, respectively, impeding IRF3 activation and type I IFN induction (146, 147). Moreover, besides affecting type I IFN induction, HSV-1 UL24 selectively restricts NF-κB promoter activation through binding NF-κB subunits p65 and p50 (148). Of note, besides HSV-1 encoded proteins, microRNA-24, which is induced by HSV-1 infection, targets the 3’ untranslated region of STING mRNA and impairs its translation (149).

### 4.2 Human papillomavirus (HPV) infections

Human papillomavirus (HPV) is a circular double-stranded DNA virus, which can cause benign and malignant lesions in humans (150–153). The study of HPV has mainly been driven by the severity of HPV-associated pathologies. HPVs are the infectious agents of benign lesions, as well as anogenital and oropharyngeal cancers. About 99% of cervical cancers are caused by the infection of high-risk HPVs (HR-HPVs) (154). There are 15 identified HR-HPV types, including HPV16, 18, 31, 33, 35, 39, 45, 51, 52, 56, 58, 59, 68, 73, and 82 (155). HPV-driven malignant transformation in cervical cancer is mainly correlated with biological processes mediated by the HPV oncogenes. HPV18 E7 has been shown to antagonize DNA sensing by using the LXCXE motif to interact with STING in cervical squamous cell carcinoma (156). Besides, HPV16 E7 is reported to interfere with cGAS-STING response in head and neck squamous cell carcinomas (HNSCCs) and oropharyngeal squamous cell carcinomas (OPSCC) (157, 158). However, the underlying mechanism through HPV16 E7 remains unknown. To figure out the mechanism, Xiaobo Luo et al. reported that HPV16 E7 in HNSCC shares low homology with HPV18 E7, and promotes autophagy-dependent degradation of STING *via* interacting with NLRX1 (159). Besides autophagy, HPV oncogenes also employ epigenetic factors to manipulate cGAS-STING response. H3K9-specific DNA methyltransferase SUV39H1 has been shown to be upregulated by HPV E7 to silence the expression of both cGAS and STING (160). In addition, HPV evolves a unique vesicular trafficking pathway to protect itself from cGAS/STING surveillance (161). Although some progress has been achieved in understanding the cGAS-STING pathway manipulated by HPV, novel mechanisms need to be illuminated. Acquiring an in-depth understanding of the cellular proteins and pathways overthrown by HPV during infection, and especially during carcinogenesis, will assist in the development of novel therapeutic agents.

### 4.3 COVID-19

Coronavirus disease 2019 (COVID-19), which is caused by severe acute respiratory syndrome coronavirus 2 (SARS-CoV-2), has spread around the world and possesses significant threats to public health worldwide. SARS-CoV-2 is a positive-sense single-stranded RNA (ssRNA). It has been demonstrated that cGAS-STING could not only recognize dsDNA from DNA viruses but also engage in RNA virus infection. The contribution in RNA virus infection involves the recognition of viral signatures or cellular DNA released from mitochondria or nuclei induced by cellular stress (162, 163). Emerging evidence suggests that cGAS-STING pathway is involved during SARS-CoV-2 infection (164–167). Intranasal administration of diABZI-4, a STING agonist, before or even after SARS-CoV-2 infection provides complete protection from severe respiratory disease in K18-ACE2 transgenic mice. Mechanistically, intranasal treatment with diABZI-4 induced the oligomerization of STING, resulting in subsequent expression of a variety of IFN-stimulated genes (ISGs), including *Cxcl10*, *interferon-induced protein with tetratricopeptide repeats 1* (*Ifit1*), *Isg15*, *Mx dynamin like GTPase 1*(*Mx1*), and *signal transducer and activator of transcription 1* (*Stat1*), as well as myeloid cells and lymphocytes activation in the lung (164). Consistently, Minghua Li et al. found that treatment utilizing diABZI-4 could restrict SARS-CoV-2 infection *via* transiently stimulating IFN signaling in primary human bronchial epithelial cells and mice (165). In addition, SARS-CoV-2 infection could induce cell fusion in a spike protein- and ACE2-dependent manner, and subsequently triggers the generation of cytoplasmic genomic DNA, which then activates cGAS-STING axis to contribute interferon and pro-inflammatory gene expression (166, 168). Targeting cGAS-STING pathway *via* diABZI-4 inhibits viral replication *in vitro* (166). Interestingly, another team showed SARS-CoV-2 activates cGAS-STING signaling in endothelial cells by provoking mitochondrial DNA release, leading to cell death and type I IFN production. Furthermore, the administration of H-151, a small-molecule STING inhibitor, significantly decreases severe lung inflammation induced by SARS-CoV-2 and improves disease outcomes. This finding uncovers an unexpected immunopathology role of type I IFNs-induced by the cGAS-STING-axis in COVID-19 (169). Most recently, Christopher J et al. have observed a significant upregulation of inflammatory cytokines, in severe COVID-19 patients and SARS-CoV-2 infected lung epithelial cells. Further study shows that such inflammatory cytokines are induced downstream of NF-κB activation, which is mediated by cGAS-STING but not RNA sensors. Pharmacological inhibition of cGAS-STING axis by utilizing STING inhibitors dampens SARS-CoV-2-mediated inflammatory gene activation *in vitro* (170). To date, some reports show that induction of type I IFNs is inadequate in autopsy samples from COVID-19 patients, and SARS-CoV-2–infected cells in culture (171). However, some reports show that type I IFNs are elevated in the lungs of COVID-19 patients (169, 172). These differences between these studies may reflect differential timing of sampling and severity of disease, which also suggests cytokine induction timing may dictate the disease outcome. Thus, it is crucial to reconsider the therapeutic potential of STING agonists or inhibitors at different SARS-CoV-2 infection stages.

As a highly transmissible viral pathogen, SARS-CoV-2 has adopted multiple strategies to restrict and evade the antiviral IFN response, which has been demonstrated by several studies. 3CL of SARS-CoV-2 interrupts K63-ubiquitin modification of STING to impair the assembly of functional STING complex, which is crucial for the induction of type I IFNs (167). SARS-CoV-2 accessory protein ORF3a has been shown to interact with STING and impair the nuclear translocation of NF-κB factor p65, leading to the downregulation of NF-κB activation, thus impeding IFN promoter activation (167). In addition, SARS-CoV-2 ORF3a has also been shown to disrupt the STING-LC3 interaction, thus impeding cGAS-STING-induced autophagy to facilitate its replication (173). Most recently, SARS-CoV-2 ORF10 is found to antagonize STING-dependent antiviral response in two ways. On one hand, ORF10 restricts STING-mediated autophagy. On the other hand, ORF10 dampens STING-dependent type I IFN activation by impairing STING oligomerization and aggregation, ER to Golgi trafficking, and functional STING complex formation with TBK1 (174). Meanwhile, SARS-CoV-2 ORF9b is reported to suppress the phosphorylation and nuclear translocation of IRF3, thus subsequently reducing the induction of type I IFNs (175).

### 4.4 AIDS

To date, HIV-1 infection is still considered a global public health issue. HIV is an RNA retrovirus that has (+) ssRNA and is dependent on reverse transcription for replication (176). It has been shown that HIV could be sensed by cGAS-STING axis, which is crucial for mediating type I IFN induction (177). Besides, activated CD4^+^ T cells utilize cGAS to sense HIV for establishing a bioactive IFN-I response, which could be potentiated by HIV accessory protein Vpr (178). Whereas IFN-I induction is thought to be lacking in HIV-1 target cells due to effective evasion mechanisms (179–181). Recently, HIV-1 nonstructural protein viral infectivity factor (Vif) is reported to inhibit type I IFN production to facilitate immune evasion. Mechanism study shows that HIV infection induces the association of Vif with SHP-1, which further promotes the recruitment of SHP-1 to STING, resulting in the inhibition of K63-linked ubiquitination of STING at Lys337 by dephosphorylating STING at Tyr162 (182). In addition, HIV Vpu impairs the cGAS-dependent type I IFN response to HIV-1 in primary CD4+ T cells (178). It has been shown that HIV, with intact capsids, is transported across the cytoplasm and through nuclear pores before integration, implying a pivotal role of HIV capsids in shielding viral DNA from cytosolic sensors (183–189). Further study shows that disrupting HIV-1 capsid formation, by utilizing capsid destabilizing small molecule PF-74, leads to a cGAS-dependent IFN response (190). In line with these, Lorena et al. found that the genetic reversal of two specific amino acid adaptations in the capsid of pandemic HIV-1(M) enables activation of cGAS and innate immune responses (181). These findings demonstrate an antagonistic role for HIV accessory proteins and capsids against the host’s innate immune response activation.

### 4.5 Hepatitis B

Viral hepatitis has long been a hot spot for its high morbidity and mortality. The main cause of death in patients with viral hepatitis is the infection of the hepatitis B virus (HBV). HBV belongs to the DNA retrovirus, carrying a dsDNA genome, the relaxed-circular DNA (rcDNA), which is repaired into a covalently closed circular DNA (cccDNA) in the nuclei of infected cells. The cccDNA then synthesizes a (+)ssRNA strand called pregenomic RNA (pgRNA) in the nucleus, which is then reverse-transcribed in the cytosol for genomic DNA replication (191, 192). STING is shown highly expressed in immortalized human hepatocyte NKNT-3/NTCP cell-derived cell clones, which are resistant to HBV, *via* induction of type III IFN (193). Most recently, STING activation is found to efficiently inhibit HBV replication through epigenetic suppression of cccDNA (194). In addition, activation of cGAS-STING pathway in both HepG2 cells and mice by dsDNA or cGAMP results in significant inhibition of HBV replication (195). Of note, in this study, the researcher artificially transfected the pHBV1.3 plasmids into the cells and mice to mimic the infection of HBV instead of using the alive HBV. In line with this finding, recently, it has been shown that hepatocytes can respond to HBV rcDNA in a cGAS-dependent manner, but not to live HBV infection (192, 196), suggesting HBV escapes the sensing of its DNAs by cGAS/STING. Verrier et al. imply the underlying escaping mechanism is that HBV infection impairs the expression of cGAS and its effector gene expression (196). Although emerging evidence suggests cGAS-STING signaling pathway in hepatocytes may inhibit HBV infection, most of them use artificial transfection of HBV DNA into cells or mice, which may not represent the live HBV infection context, thus raising the concern for data authenticity. Therefore, more *in vivo* studies using live HBV infection need to be conducted to uncover the role of cGAS-STING pathway in HBV infection.

### 4.6 Flu

Influenza is an infectious respiratory disease, which is mainly caused by influenza A virus (IAV) and influenza B virus (IBV) in humans. Influenza virus impacts the respiratory tract by direct viral infection (197). Christian et al. reported that the production of interferon is impaired in STING-deficient THP-1 cells, but not in cGAS-deficient THP-1 cells after IAV infection, suggesting that IAV initiates a STING-dependent, cGAS-independent pathway, which is critical for full interferon production. To counteract the host’s antiviral defense, IAV utilizes FP (the fusion peptide), which is highly conserved among IAV and IBV strains, to impair STING dimerization and TBK1 phosphorylation in response to membrane fusion through binding STING in the region of the dimerization interphase (198). In addition, it has been shown that cytosolic mitochondrial DNA (mtDNA) plays important roles in cGAS-mediated antiviral immune responses after infection with RNA viruses, such as dengue virus and lymphocytic choriomeningitis virus (LCMV) (199–201). In the influenza viral infection context, Miyu et al. found that influenza virus M2 protein, a proton-selective ion channel, which is critical for the viral uncoating during viral entry and budding, triggers cytosolic mtDNA release in a MAVS-dependent manner, and activates cGAS- and DDX41-mediated IFN-β production. To evade host immunological surveillance, the influenza virus employs nonstructural protein 1 to interact with mtDNA to evade the STING-dependent antiviral immunity. Of note, cGAS deficiency did not markedly alter the viral titer in the lung. By contrast, STING deficiency significantly elevated the viral titer in the lung compared to that from WT mice. These findings suggest the presence of other DNA sensors that activate redundant signaling pathways to limit viral replication (202).

## 5 cGAS-STING in bacterial infectious diseases

Besides viral DNA, cGAS is reported to recognize bacterial DNA and synthesize cGAMP to activate downstream STING (203–209). Furthermore, stimuli other than cGAS induced-cGAMP, including cyclic dinucleotides (CDNs) [cyclic diGMP, cyclic diAMP], and ER stress, which could be induced by bacterial infection, can also activate STING (210–212). All these suggest the intricate interplay between bacterial infection and cGAS-STING responses. Of note, the activation of cGAS-STING signaling pathway has pleiotropic roles in the bacterial infection process.

*Chlamydia trachomatis* (*C. trachomatis*) is a gram-negative bacterium. *C. trachomatis* infection causes the most common sexually transmitted disease, with serious complications such as pelvic inflammatory disease, infertility, and ectopic pregnancy in women, and epididymitis and orchitis in men (213). It is well known that type I IFN is pivotal in antiviral response. However, the interplay between type I IFNs and bacterial infections seems somewhat pleiotropic and contexts dependent (214). Type I IFN signaling has been shown to exacerbate host pathology during *C. trachomatis* infection. It is reported that cGAS is recruited to the chlamydial inclusion membrane and mediates the generation of cGAMPs, which subsequently initiate STING-induced IFN-β expression during *C. trachomatis* infection. Moreover, the colocalization of cGAS and STING on the cytosolic side of the chlamydial inclusion membrane suggests that chlamydial DNA seems to be recognized outside the inclusion. This study also provides evidence that cGAMPs could transfer from infected cells to adjacent cells and thus upregulate IFN-β expression in adjacent uninfected cells during *in vivo* infection, escalating the pathogenesis (215). On the contrary, a recent *in vivo* study shows that mice deficient in either cGAS or STING significantly increased the yields of live *C. trachomatis* in the lower genital tract, demonstrating a beneficial role of cGAS-STING response during *C. trachomatis* infection (216). Moreover, STING also engages in the induction of type I IFN response *via* the detection of bacterial metabolite cyclic di-AMP during *C. trachomatis* infection (217). Further study shows that such type I IFN is essential for inflammasome activation (218). These studies suggest STING/interferon pathway may be a potent therapeutic target to treat *C. trachomatis* infection and its associated inflammatory pathology. *Staphylococcus aureus* (*S*. *aureus*) is a gram-positive bacterium that is the leading cause of infective endocarditis, as well as skin, soft tissue, and pleuropulmonary infections (219). *S*. *aureus* DNA is demonstrated to be sensed by cGAS. Recently, cGAS is identified as a DNA sensor for recognizing the extracellular pathogen *S*. *aureus* and *Pseudomonas aeruginosa* (*P. aeruginosa*) (a gram-negative bacterium), inducing the production of type I IFNs. Though the cGAS-STING-IFN axis is shown to be critical for restricting the *P. aeruginosa* infection, the biological function of this type I IFN in *S*. *aureus* replication remains unclear in this study (209). Intriguingly, Casey et al. report cyclic di-AMP, which is released extracellularly from *S. aureus* biofilm, induces a STING-dependent type I IFN response in macrophages, facilitating *S. aureus* intracellular survival in macrophages (220). Since type I IFNs could induce the activation of STAT3, which is a key mediator of anti-inflammatory signaling (221), the cGAMPs-STING-type I IFNs axis may be the underlying mechanism, through which biofilms render the host an anti-inflammatory state, thereby resulting in the persistent biofilm-mediated infections in an immunocompetent host (222). Moreover, STING and TLR pathways are activated at the early stage of infection with live *S. aureus*. However, they display opposite roles in host defense to *S. aureus*. On one hand, IL-1β induction *via* TLR signaling protects the host against *S. aureus* infection. On the other hand, type I IFNs induced by STING signaling could inhibit the transcription of IL-1β as well as the processing of pro-IL-1β protein into mature IL-1β through the inflammasome. The orchestrated immune response operated by TLR and STING signaling contributes to establish an effective anti-microbe response, meanwhile, protects the host from overwhelming inflammatory response (223). In addition, STING was shown to enhance host defense against *S. aureus* infection *via* blocking the necroptosis of macrophages (224), although previous studies show that cGAS-STING contributes to the necroptosis in macrophages in a type I IFN-dependent manner (225, 226), suggesting the specific role of cGAS-STING in distinct contexts. *Listeria monocytogenes* (*L. monocytogenes*) is a gram-positive intracellular foodborne pathogen and the causative agent of listeriosis (227, 228). The innate immune system is a double edge sword, that is it not only may contribute to defending against infection, but also could lead to pathology. DNA from *L. monocytogenes* is sorted into EVs in infected cells through a STING-TBK1-MVB12b (multivesicular body protein) pathway and delivered to bystander cells to stimulate cGAS-STING pathway, which facilitates the spread of infection signs across tissues prior to the actual infection process. Moreover, the EVs from infected macrophages could promote FasL-stimulated apoptosis of splenic T lymphocytes, which is also dependent on the presence of cGAS and STING in the T lymphocytes, suggesting the exquisite immune evasion strategy employed by *L. monocytogenes* (229). It is reported that type I IFN, which is induced by *L. monocytogenes* infection is deleterious to the host’s anti-bacterial response *via L. monocytogenes*-mediated apoptosis of leukocytes (230–232). The complement anaphylatoxins, C5a and C3a that are generated during activation of the complement cascade in response to infection are shown to inhibit the production of detrimental IFN-β by damping the expression of DEAD-box helicase 41 (DDX41), STING, phosphorylated TBK1, and phosphorylated p38 mitogen-activated protein kinase (MAPK), which play critical roles in the type I IFN induction upon *L. monocytogenes* infection (233). Interestingly, Alexander et al. reported that *L. monocytogenes-*derived c-di-AMP activated STING and induced type I IFN signaling. Deficiency of STING decreased the influx of inflammatory monocytes and increased systemic bacterial burden during enterocolitis (234). In addition, STING deficiency impaired the Ly6C^hi^ monocytes, increasing bacterial burden in the liver during *L. monocytogenes* infection (235). *Streptococcus pneumoniae* (*S. pneumoniae*) is a leading cause of pneumonia, bacteremia, meningitis, and otitis media (236). *S. pneumoniae* produces c-di-AMP *via* CdaA, a diadenylate cyclase (237). And phosphodiesterase 1 (Pde1) and Pde2 are responsible for the degrading of c-di-AMP (237). Pneumococci deficient in Pde2 led to increased concentrations of c-di-AMP in the mutant pneumococci and resulted in the hyperactivation of STING and excessive IFN-β expression, as well as rapid cytotoxicity (238). However, another study showed that cGAS-STING pathway had no contribution to the immune response against *S. pneumoniae* in mice and humans, although pneumococcal DNA could be detected by cGAS and then initiated type I IFN production through STING (207). Moreover, it is reported that STING promoted coagulation in a type I IFN response-independent manner, increasing the risk of severe sepsis caused by *S. pneumoniae* (239). In addition to type I IFN, IFN-γ could also be induced upon *S. pneumoniae* infection. Elevated plasma IFN-γ was correlated with increased mortality in patients with *S. pneumoniae* sepsis (240). Deficiency of IFN-γ rendered mice more resistant to developing pneumococcal meningitis (241). It has been shown that cGAS-STING pathway cooperates with MyD88 pathway in Ly6C^hi^ monocytes to promote late-stage lung IFN-γ production *via* the production of IL-12p70 during pulmonary pneumococcal infection (242). *Mycobacterium tuberculosis* (*M. tuberculosis*) is the etiological agent of tuberculosis (TB) and the leading cause of death due to its transmissible and drug-resistant features (243). *M. tuberculosis* has been shown to initiate the cytosolic DNA surveillance pathway *via* phagosome disruption mediated by the mycobacterial protein secretion system ESX-1 (244). Multiple reports showed that the exposed mycobacterial DNA in the cytosol *via* ESX-1 system could be sensed by cGAS-STING pathway and activate type I IFN production and autophagy process, suggesting an unanticipated cross-talk between DNA surveillance and autophagy, meanwhile indicating a major role for selective autophagy process in resistance to *M. tuberculosis* infection (204, 206). On the contrary, *M. tuberculosis* has adopted delicate strategies to evade host DNA surveillance to protect its survival (245–247). For instance, *M. tuberculosis* coding protein Rv0753c (MmsA) has been shown to interact with STING and promote STING for p62-mediated autophagic degradation, thus damping STING-mediated type I IFN production (245). Besides, *M. tuberculosis* phosphodiesterase (PDE) CdnP was identified to restrict STING activation and the type I IFN response through hydrolysis of both bacterial-derived c-di-AMP and host-derived cGAMP (247). Due to the severity and infectivity of TB, although CDNs-adjuvanted protein subunit vaccine (248) and the inhibitor, that targeting *M. tuberculosis* PDE (246) can protect the host from *M. tuberculosis* infection, there is a lot of room remaining to uncover to pave the way for TB therapy.

## 6 cGAS-STING in fungal infectious diseases

Fungi and mammals share a co−evolutionary history and are involved in a complex web of interactions (249). Superficial and invasive fungal infections lead to diseases that range from irritating to life-threatening, developing invasive infections during their lives, and mortality for these infections often exceeds 50% (250). Antifungal immunity is mainly composed of cellular innate-, adaptive-, humoral-, and mucosal-immune mechanisms. And considerable PRRs, like TLRs, NOD2, Dectin families, and CLR families have been proven momentous for the detection of fungal PAMPs (249, 251–253). However, the role of cGAS-STING signaling acting on antifungal immunity is less known. Although Majer et al. reported that type I IFN induced by *Candida albicans* infection promotes sepsis as type I IFN recruits and activates inflammatory monocytes/DCs in a CCL2-dependent way, which causes high host-destructing potency, the mechanism of type I IFN production and whether it depends on cGAS-STING are still ill-defined (254). Besides, β-Glucans, the major cell wall structural components in fungi, have been applied to anti-tumor research. Kalafati et al. found β-glucan-induced trained immunity was related to transcriptomic and epigenetic rewiring of granulopoiesis and neutrophil reprogramming toward an anti-tumor phenotype, which is type I IFN dependent (252, 255). Given the unequivocal function of cGAS-STING as a bridge to link pathogens’ PAMPs and type I IFN responses, we have reasons to believe cGAS-STING possesses a potentially important role in antifungal immunity, which would be interesting to comprehend the physiological function and underlying mechanism of the cGAS-STING pathway in the fungi infection.

## 7 Conclusion and perspectives

In the past few years, we have obtained a mounting knowledge of the structures and biomedical functions in cGAS-STING pathway, which provides a new framework for understanding the immune stimulatory capacity of dsDNA and c-GAMPs. The exquisite sensing of the pathway to foreign nucleic acids and CDNs enables the development of robust anti-microbe responses to protect the host from invading pathogens. Whereas, due to the pleiotropic biological functions of activation of cGAS-STING pathway and type I IFNs as well as inflammatory cytokines in distinct infection contexts, continuing efforts will need to obtain more in-depth knowledge on the effects of the cGAS-STING pathway in infectious disease pathogenesis in specific scenarios. These efforts will advance side by side with *in vivo* studies by using conditional knockout mice or in *ex vivo* studies by using human samples. Furthermore, emerging evidence from functional and mechanistic studies suggests that multiple intracellular and extracellular information, such as pathogen DNA contained-EVs, mitochondrial DNA release, ER stress, and activation of other signaling pathways, cooperates with cGAS-STING to initiate an efficacious detection of invading pathogens and subsequent prompt activation of innate immune signaling. However, the underlying mechanism of manipulating cGAS-STING pathway to avoid aberrant and excessive immune response is still not fully understood. In this regard, more efforts need to be put into the regulation mechanism of cGAS-STING pathway on molecule and cell scales. Accordingly, to avoid the deleterious consequences of aberrant activation of cGAS-STING pathway, usage of agonists or antagonists of cGAS and STING in certain infection contexts may be instructive. As we discussed above, cytokine induction timing may dictate the disease outcome. Thus, it is crucial to revalue the therapeutic potential of agonists or antagonists at different infection stages. Moreover, the hallmark of the interplay between innate immune response and infectious microbes is the pathogen evasion process. Various counteraction mechanisms involved in cGAS-STING pathway have already been elucidated. However, how pathogen factors (pathogen proteins, nucleic acids, and metabolites) interact with cGAS and STING, and how pathogen factors manipulate the structure and signal transduction of this innate immune pathway are still not fully understood. New insight into evasion strategies employed by pathogens would deepen our knowledge of infectious disease pathogenesis, thereby providing new opportunities for developing therapeutic interventions.

## Author contributions

R-FW, XY, YD, ZH and YL designed and wrote the manuscript. YD, ZH, YL, HW, XY, and R-FW discussed and revised the manuscript. All authors contributed to the article and approved the submitted version.
